# Sealing Performance Analysis for the Flange Joint at the Front End of a High-Power Diesel Engine Exhaust Manifold

**DOI:** 10.3390/ma19183830

**Published:** 2026-09-09

**Authors:** Qinggele Hua, Jing Ding, Wenjie Qin

**Affiliations:** School of Mechanical Engineering, Beijing Institute of Technology, Beijing 100081, China

**Keywords:** sealing performance, contact stress, creep, bolted flange joint

## Abstract

**Highlights:**

• An experiment was designed to test the sealing performance of the flange joint.
• The contact stresses on the gasket under the critical bolt preload were obtained.
• The effect of material creep on the sealing performance of the joint was investigated.

**Abstract:**

The front end of the exhaust manifold in a high-power diesel engine is connected to the cylinder head with a bolted flange joint. The high-temperature gas discharged from the cylinder poses a great challenge to the sealing performance of the flange joint. In this paper, in order to provide a predictive foundation for the sealing design of the flange joint at the front end of a high-power diesel engine exhaust manifold, the finite element (FE) analysis was implemented to determine the contact stress distributions on the joint surfaces under the high-temperature working condition. An experiment was designed and conducted to obtain the contact stresses on the gasket in the testing flange joint under the critical bolt preload needed to meet the leakage rate requirement, which were used to compare with the contact stresses on the gasket in the actual flange joint to evaluate the sealing performance of the actual flange joint. The results show that the sealing performance of the actual flange joint is qualified. Considering the effect of material creep deformation on the sealing performance of the flange joint, the creep analysis was conducted based on the creep constitutive models of the materials obtained through creep tests, and the continuous sealing time of the flange joint at the high working temperature was predicted. This approach of combining numerical simulation with experimental testing can also be used for sealing prediction and design of other bolted flange joints working at high temperatures.

## 1. Introduction

The temperature of the gas emitted by a high-power diesel engine can reach 600 °C or even higher [1,2,3], so the exhaust manifold is subjected to extremely high thermal loads. The front end of the exhaust manifold is connected to the cylinder head with a bolted flange joint. If the sealing of the joint is poor, high-temperature exhaust gas may leak from here and cause local overheating, which is not conducive to the thermal management of the power system.

Bolt flange joints are widely used detachable sealed connections in pressure containing equipment, such as pressure vessels and pipes [4,5,6,7]. The evaluation of the sealing performance of bolted flange joints generally focuses on two indicators; one is the stresses on the contact surfaces [8,9,10,11,12,13,14,15]. In a study, Abi et al. computed the stresses of a gasketed bolted flanged pipe joint using 3D nonlinear finite element (FE) analysis considering the combinations of internal pressure, axial and thermal loading, and the sealing performance of the joint was evaluated based on whether the stresses on the gasket were higher than the minimum required seating stress recommended by the manufacturer [8]. Aljuboury et al. studied the contact stresses on the aluminium gasket in a bolted steel flange joint using ANSYS v18 software, and determined where the fluid leakage propagation would occur on the contact surface by comparing the contact stress and the fluid pressure, which was modelled by a pressure-penetration criterion using the contact element real constant PPCN. If the contact stress was less than the fluid pressure at a point, the fluid started to penetrate from the starting point towards the outside [9]. Unlike using numerical calculations, some researchers used a non-invasive ultrasonic technique to quantify the distribution of contact stress [16]. Some researchers have investigated the effect of high-temperature creep of materials on stresses [17,18,19,20]. Another sealing performance indicator is the leakage rate, which is a phenomenon that cannot be prevented completely in a joint and is also closely related to contact stresses [21,22,23,24,25,26]. Therefore, achieving accurate contact stresses on the contact surfaces is necessary for analyzing the sealing performance of flange joints. However, the critical contact stress required for sealing bolted flange joints varies greatly with working conditions, and its value is often provided by manufacturers without a universally applicable design criterion. In this study, an experiment was designed to determine the critical bolt preload required to meet the leakage rate requirement of the flange joint at the high working temperature and then obtain the critical minimal contact stress on the gasket in the testing flange joint through FE analysis, since the manufacturer had not provided a minimum required stress for the sealing design. This critical minimal contact stress was used to compare with the contact stresses on the gasket in the actual flange joint to evaluate the sealing performance of the actual flange joint.

At a joint of a bolt flange, a gasket is usually fitted between the two flange surfaces to compensate for surface micro unevenness and provide high tightness [27,28]. Its deformation in the thickness direction, which is often represented by a compression–springback curve, has a significant impact on the contact stresses on flange joint surfaces [10,29,30,31]. However, the compression–springback characteristics of gaskets in most studies are obtained through compression experiments or numerical calculations under room temperature or low-temperature conditions, with little consideration for high-temperature conditions. In this study, considering the high working temperature of the gasket at the front end of the flange joint in the diesel engine exhaust manifold, a combination of experimental testing and numerical simulation was utilized to obtain the compression–springback curve of the gasket at the high temperature to accurately simulate its compression–springback characteristics and obtain the contact stress distributions at the working temperature.

The sealing of a flange joint relies on the contact stress established at the joint interface by bolt preload. Under long-term high-temperature working conditions, the parts in the joint will undergo creep deformation, leading to the relaxation of preload [32,33,34] and a decrease in contact stress at the joint surface [17,18,19,20]. Due to the fact that creep deformation is related to factors such as material, stress and temperature, and the influence of part material creep on bolt flange joint sealing is also related to the structure and specific working conditions of the joint, previous research results cannot be fully applicable to the studied flange joint. Therefore, further investigation was implemented on the effect of the high-temperature creep of the part materials on the sealing performance of the flange joint.

## 2. Cylinder Head–Gasket–Exhaust Manifold Assembly

### 2.1. Structure of the Assembly

**Figure 1:**
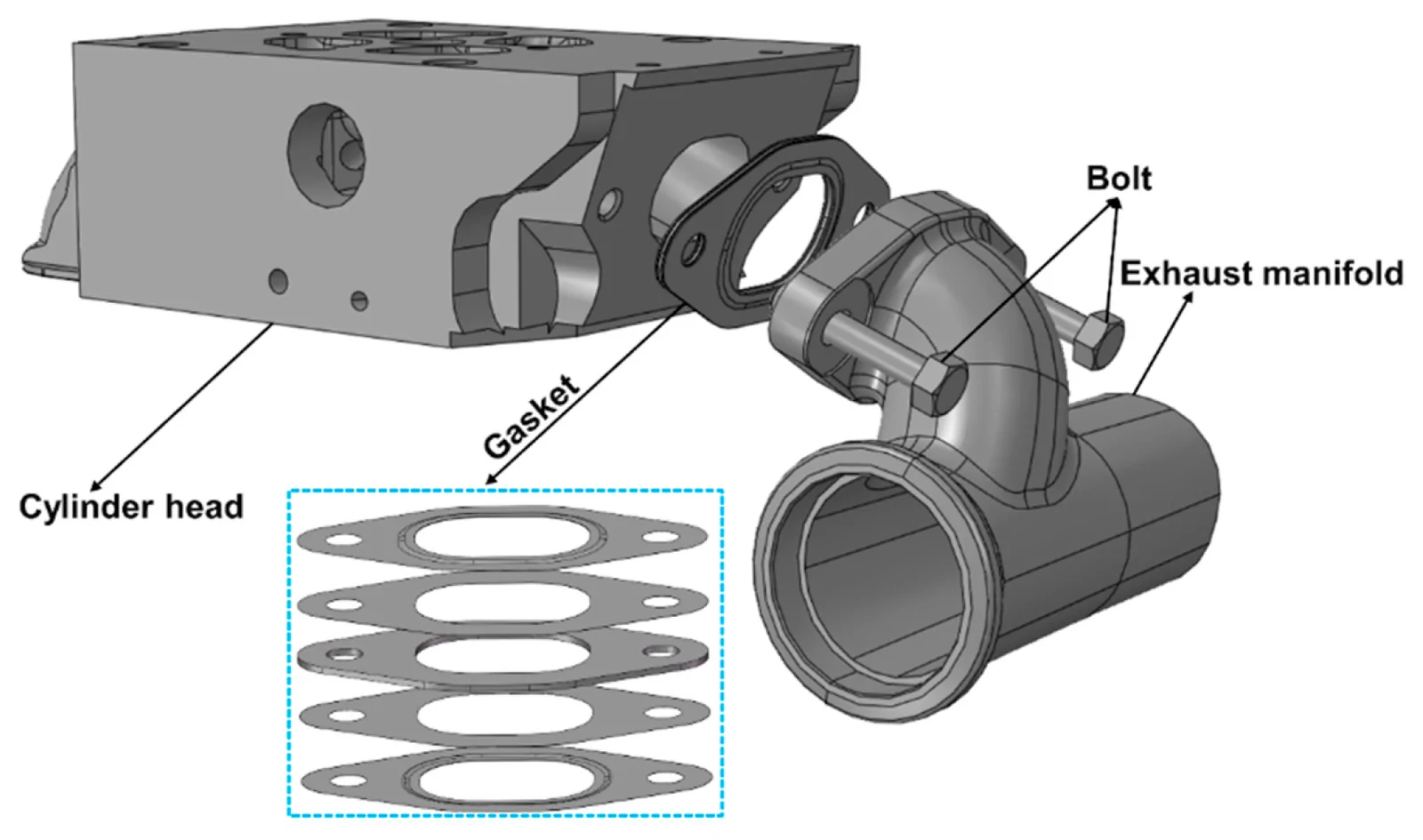
Structure of the cylinder head–gasket–exhaust manifold assembly.

Figure 1 shows the assembly of the cylinder head, the gasket and the exhaust manifold for one cylinder of a high-power diesel engine. The metal gasket consists of five layers. The upper two layers and the lower two layers of the gasket are made of a high-nickel alloy, with each layer being 0.1 mm thick. The central layer of the gasket is made of 06Cr19Ni10 stainless steel and is 1.6 mm thick. There is a ring of convex ribs with a width of 2 mm on the top and bottom layers of the gasket, which are called sealing rings here. The gasket is installed between the exhaust manifold flange and the cylinder head flange and is fastened together by two bolts.

### 2.2. Temperature Field Distribution

**Figure 2:**
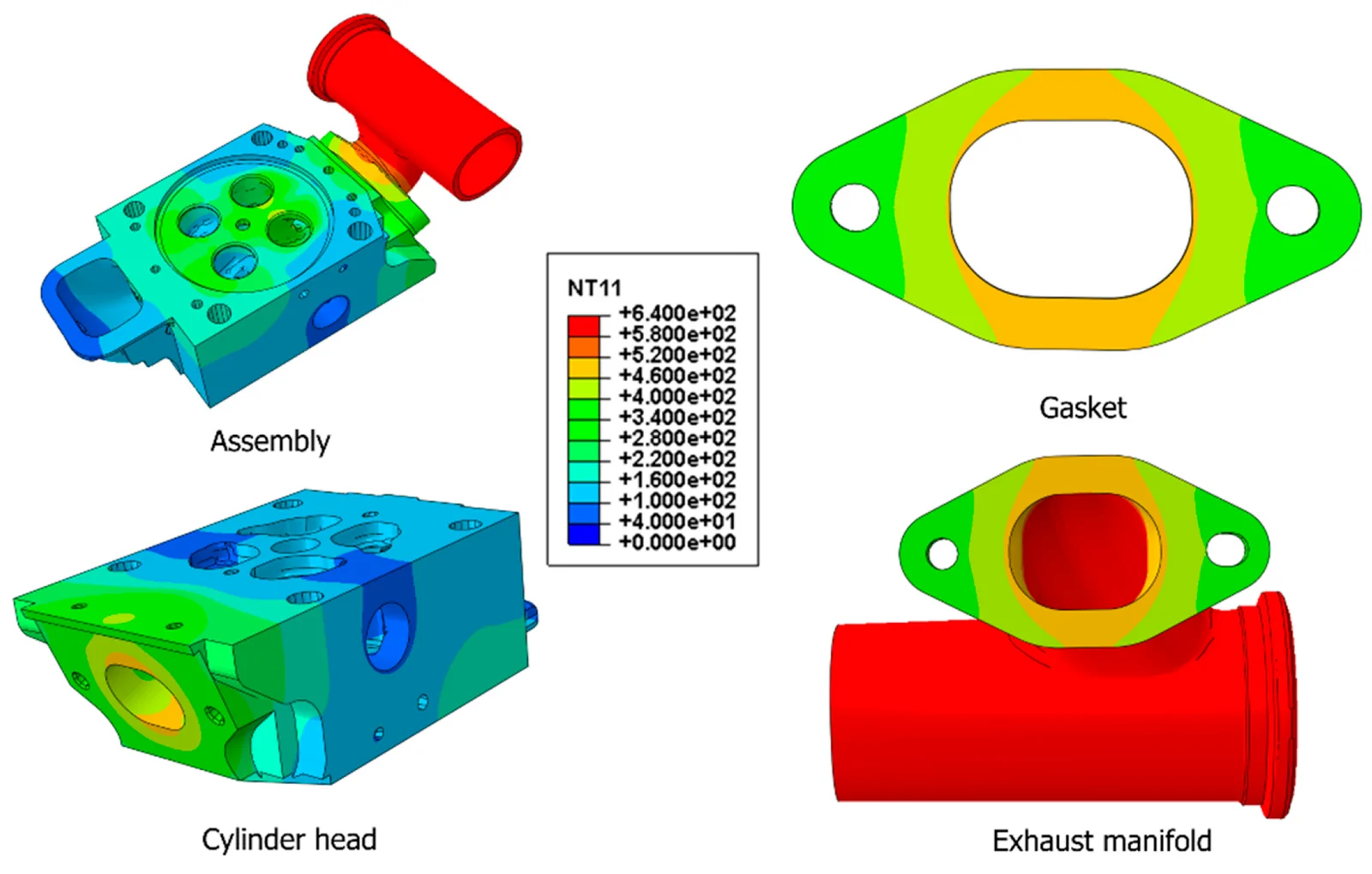
Temperature distributions of the assembly and the parts.

In this study, the temperature field distribution of the assembly was first obtained through steady-state thermal analysis. The thermal boundary conditions include the temperature values and heat transfer coefficients of different regions, as shown in Table 1, and the contact thermal conductivity of contact pairs of 10,000 W·m^−2^·K^−1^, which were provided by the manufacturer. As shown in Figure 2, the temperature distributions on the contact surfaces of the three parts in the flange joint are similar, and the temperature in the regions near the gasket sealing ring is around 500 °C.

## 3. FE Analysis of Stress

### 3.1. FE Model

**Figure 3:**
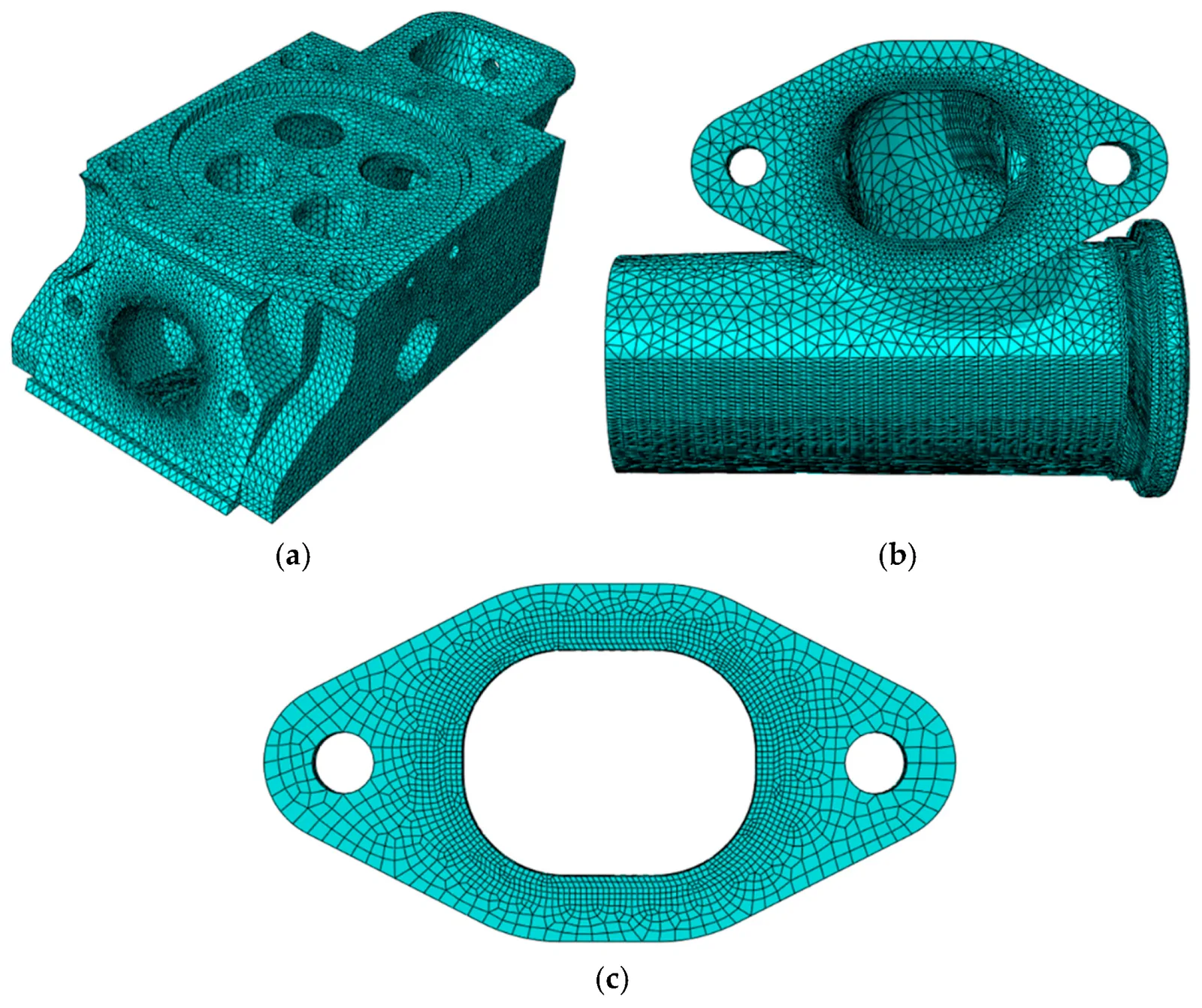
FE mesh model: (**a**) cylinder head, (**b**) exhaust manifold and (**c**) gasket.

Figure 3 shows the FE mesh model obtained by meshing the geometric model presented in Figure 1. The parts with complex geometric structures, including the cylinder head and the exhaust manifold, were discretized using second-order 3D stress elements with a global element size of 4 mm. The gasket was meshed by the sweeping method using second-order gasket elements generated with a global element size of 3 mm and their deformation along the thickness direction is defined by the compression–springback curve at 500 °C obtained in Section 3.3. The effect of the sealing ring height was simulated by defining the properties with different thicknesses. The mesh of the gasket sealing ring and the areas in contact with the sealing ring on the cylinder head flange and the exhaust manifold flange surfaces were refined, and the element size was determined to be 1 mm by the convergence analysis. Table 2 gives the variation in the minimal contact stress on the gasket sealing ring with the element size. It can be seen that the contact stress value has basically converged when the element size is less than 1.5 mm.

Contact pairs were defined between the cylinder head, the gasket and the exhaust manifold where the friction coefficient between the contact surfaces was set to be 0.19. The penalty function method was applied for contact analysis, in which the normal non-penetrability condition (1) and the tangential constraint condition (2) must be satisfied at each contact point of the interface.

(1) *σ*_N_*g*_N =_ 0

(2) $$∥\sigma_{T}∥+\mu \sigma_{N}\leq 0$$

where *σ*_N_ is the normal contact stress, *g*_N_ is the penetration distance between contact nodes, *σ*_T_ is the tangential contact stress, and *μ* is the friction coefficient.

The bolts were modeled using beam elements, and the coupling constraints of translational degrees of freedom were applied between the top nodes of the beams and the nut seating surfaces on the manifold flange, and between the bottom nodes of the beams and the threaded hole surfaces in the cylinder head. Displacement constraints were applied on the areas of the bottom surface of the cylinder head in contact with the cylinder block, and a designed bolt preload of 25,000 N was applied to each bolt. Finally, a gas pressure of 0.5 MPa was applied to the inner wall of the exhaust pipe, and the temperature field obtained in Section 2.2 was applied to the entire structure.

### 3.2. Material Properties

**Figure 4:**
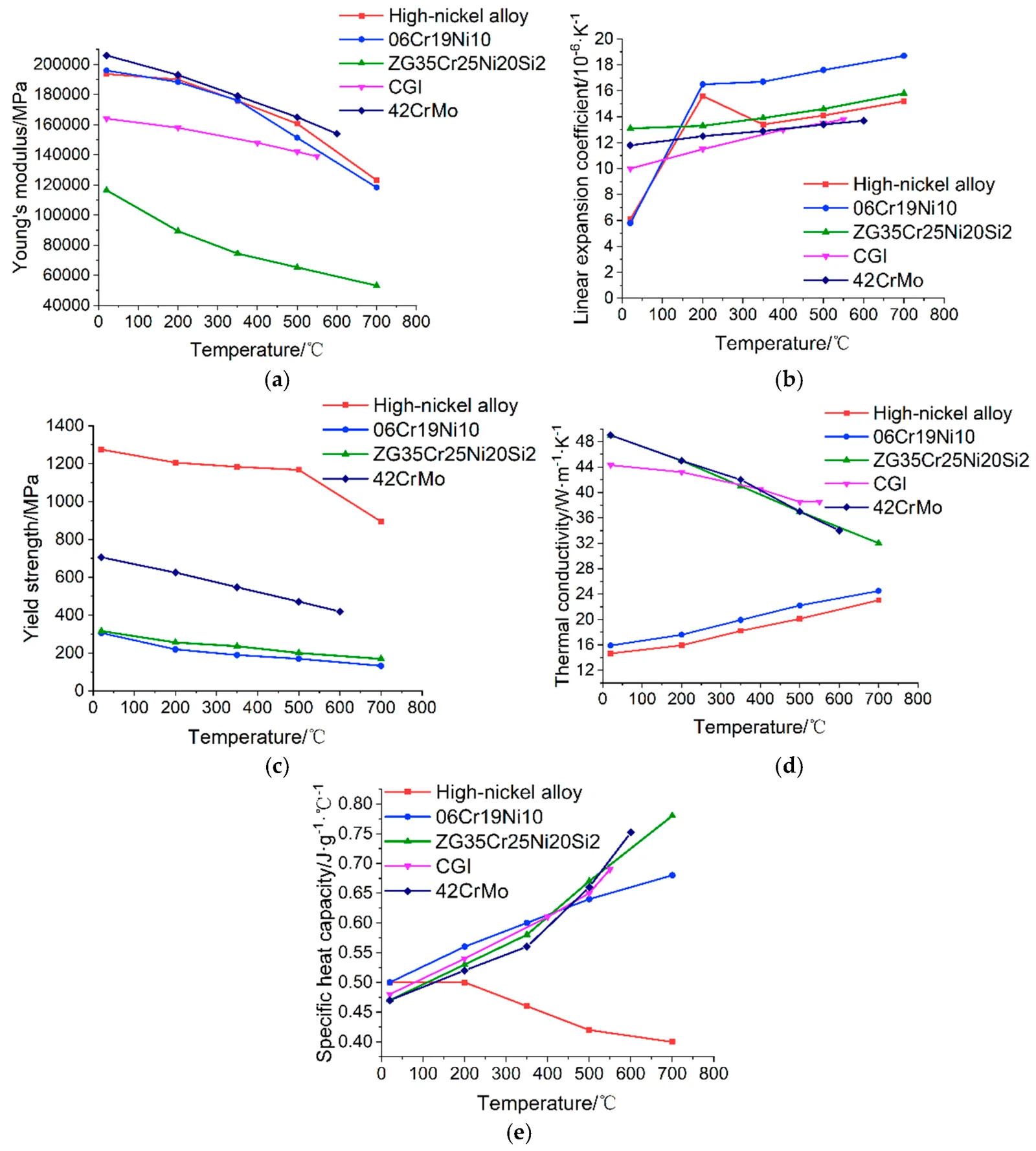
Material properties: (**a**) Young’s modulus, (**b**) coefficient of linear thermal expansion, (**c**) yield strength, (**d**) thermal conductivity and (**e**) specific heat capacity.

The cylinder head is made of compacted graphite cast iron (CGI), the exhaust manifold is made of ZG35Cr25Ni20Si2, the bolts are made of 42CrMo, and the gasket is composed of four high-nickel alloy layers and one 06Cr19Ni10 stainless steel layer. The material properties were experimentally measured, and the material constitutive models were all based on ideal elastic–plastic models. The uniaxial tensile tests were adopted to determine the stress–strain relationships, Young’s moduli and the yield strengths. Differential scanning calorimetry was adopted to measure the specific heat capacities, and the thermomechanical analysis was adopted to determine the coefficients of linear thermal expansion. The thermal conductivities were obtained using the steady-state heat flux method. The temperature-dependent material properties are presented in Figure 4. The yield strength of the cylinder head material was measured to be 150 MPa at room temperature and 125 MPa at higher temperature.

### 3.3. Compression–Springback Curves of the Gasket

**Figure 5:**
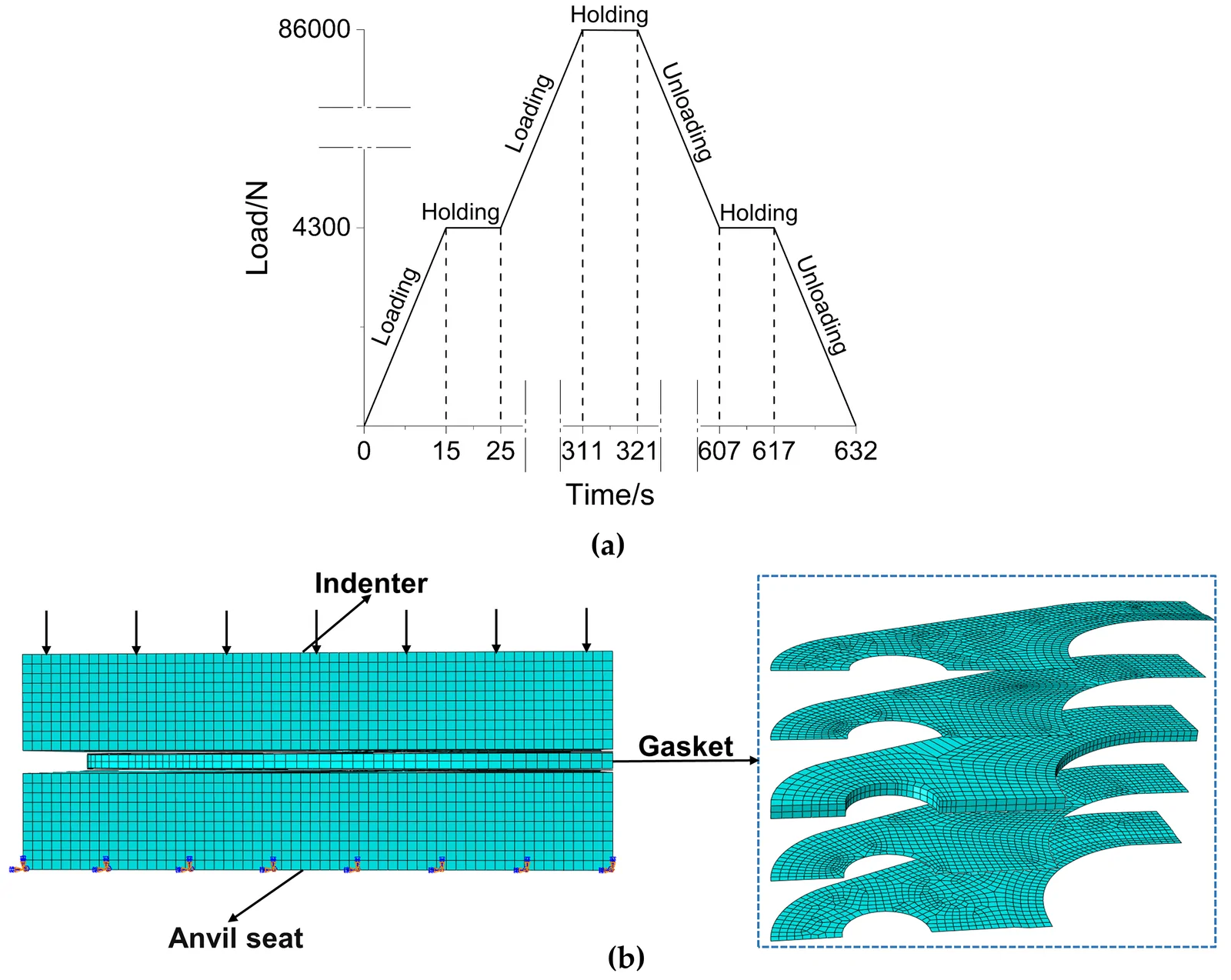
Compression–springback behavior test machine and simulated model: (**a**) time-varying load and (**b**) FE model.

**Figure 6:**
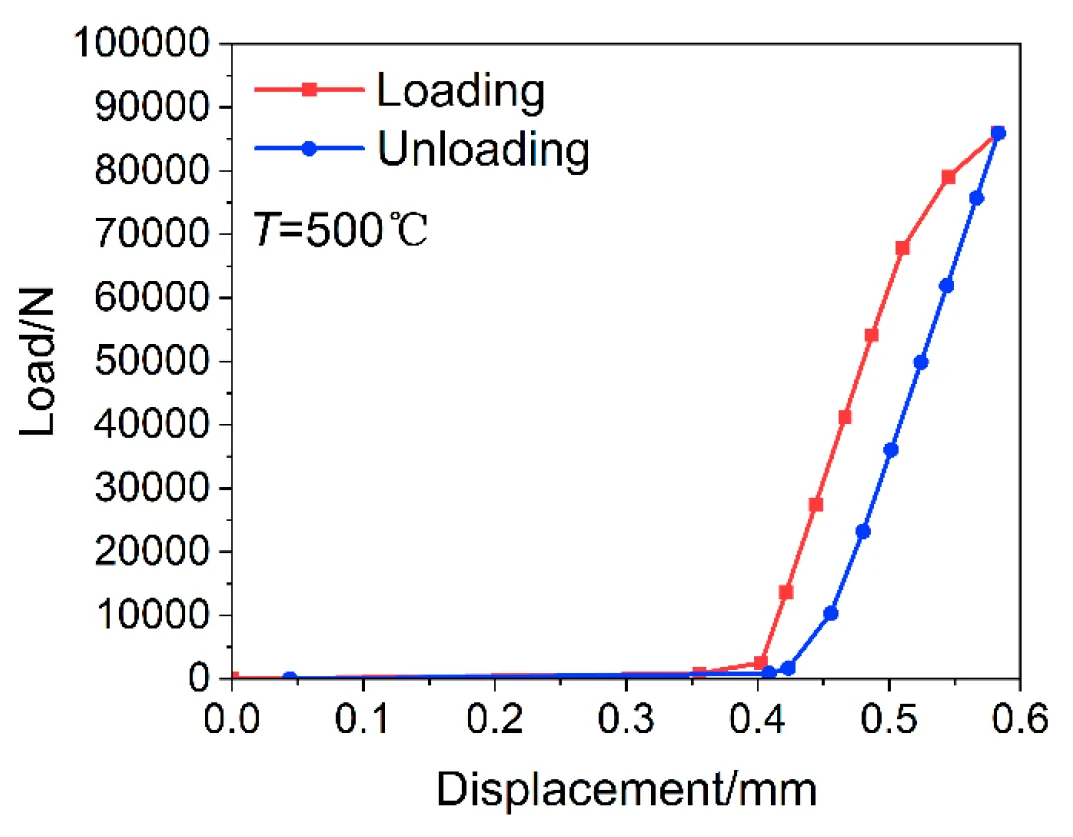
Compression–springback curve of the gasket at 500 °C.

The compression–springback curve of the gasket is essential for accurate contact stress distribution calculations when modeling the gasket. In this study, the numerical simulation with experimental verification was used to study the compression–springback characteristics of the multilayer metallic gasket. Firstly, compression–springback curves at room temperature (20 °C) and 200 °C were experimentally measured by the compression test using a universal testing machine. The loading process used is shown in Figure 5a. Subsequently, an FE model simulating the compression test setup, including a quarter of the multi-layer metal gasket, the anvil seat and the indenter (see Figure 5b), was developed considering symmetry. A time-varying load as shown in Figure 5a was applied to the top surface of the indenter, a fixed constraint was applied to the bottom surface of the anvil, and surface-to-surface contacts were established between the contact surfaces. The FE dynamics analysis was implemented to simulate the compression–springback processes. The compression–springback curves at room temperature and 200 °C were obtained and compared with the corresponding experimental curves as shown in Figure S1a,b to verify the accuracy of the model. Furthermore, the FE model was utilized to calculate the compression–springback curve at 500 °C, which is shown in Figure 6. More discussion about compression–springback curves of the gasket under different temperatures is given in the Supplementary Information. Comparing the compression–springback curve at 500 °C with the curves at 20 °C and 200 °C in the Supplementary Information, it can be found that the slope of the curve at 500 °C is smaller and the separation between the loading curve and the unloading curve is significantly larger. Therefore, the compression stiffness of the gasket will decrease and the inelastic deformation will increase at high temperatures, which will affect the accuracy of the simulated contact stress results at high temperatures.

### 3.4. Contact Stress Results

**Figure 7:**
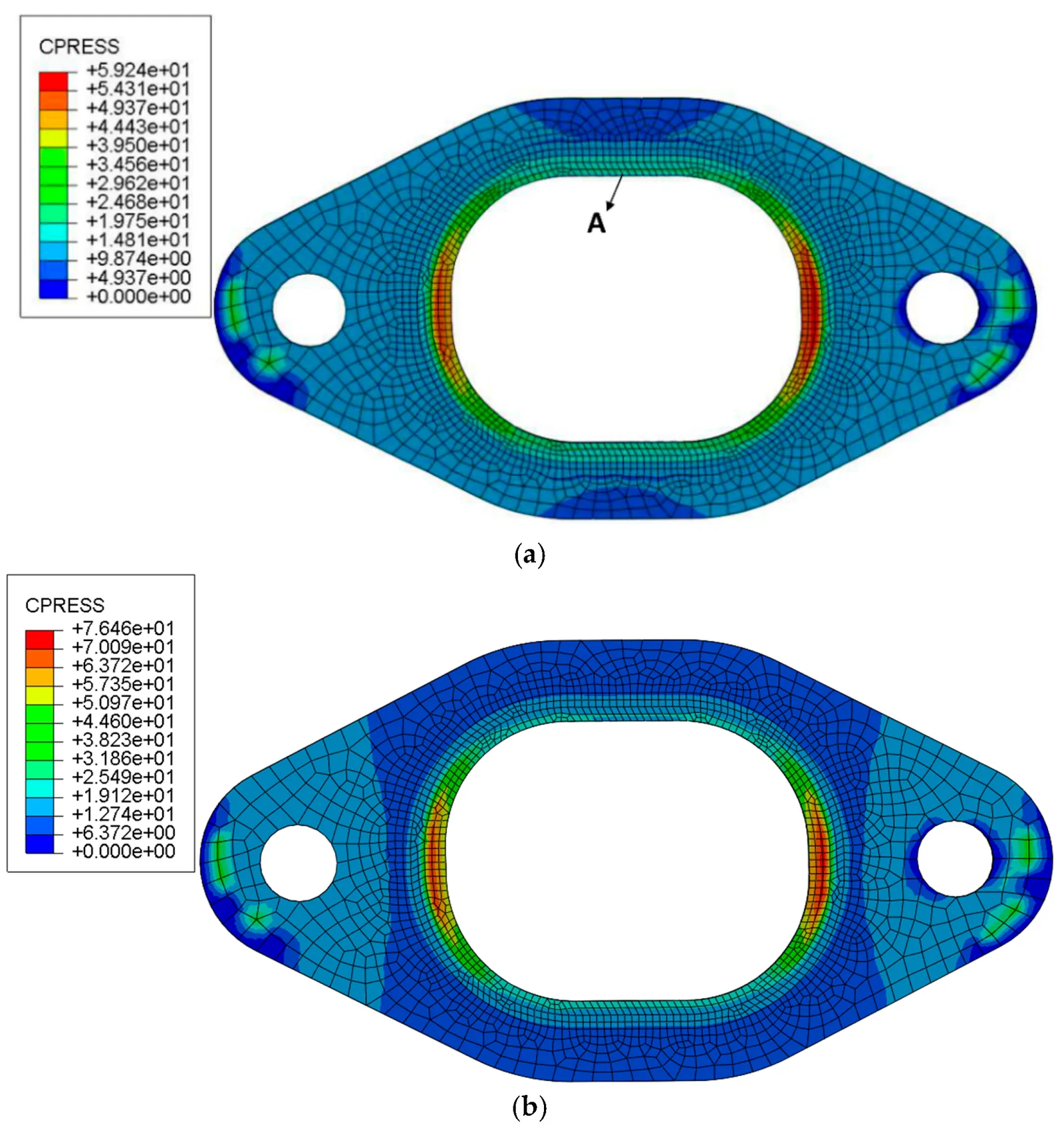
Contact stress distributions on the gasket at different temperatures: (**a**) room temperature and (**b**) 500 °C.

The FE model of the cylinder head–gasket–exhaust manifold assembly established in Section 3.1 was utilized in the thermal stress analysis with the introduction of the node temperatures in Section 2.2. The contact stresses were obtained to evaluate the sealing performance of the flange joint. The resulting stress distributions on the contact surfaces of all three parts are similar, and the contact stress distributions on the gasket at room temperature and high working temperature are shown in Figure 7. It can be seen that the contact stresses on the longer sides of the gasket sealing ring are smaller than those on the shorter sides, so gas leakage is more likely to occur on the longer sides, which is consistent with the actual observations. The minimum contact stress at the edge of the sealing ring is located at Point A in Figure 7a with a value of 19.7 MPa at room temperature, which slightly decreases to 19.0 MPa at 500 °C. The effects of height and width of the sealing ring on the contact stresses are given in the Supplementary Information as shown in Figure S2.

## 4. Sealing Test and Evaluation of the Flange Joint

### 4.1. Testing Setup

**Figure 8:**
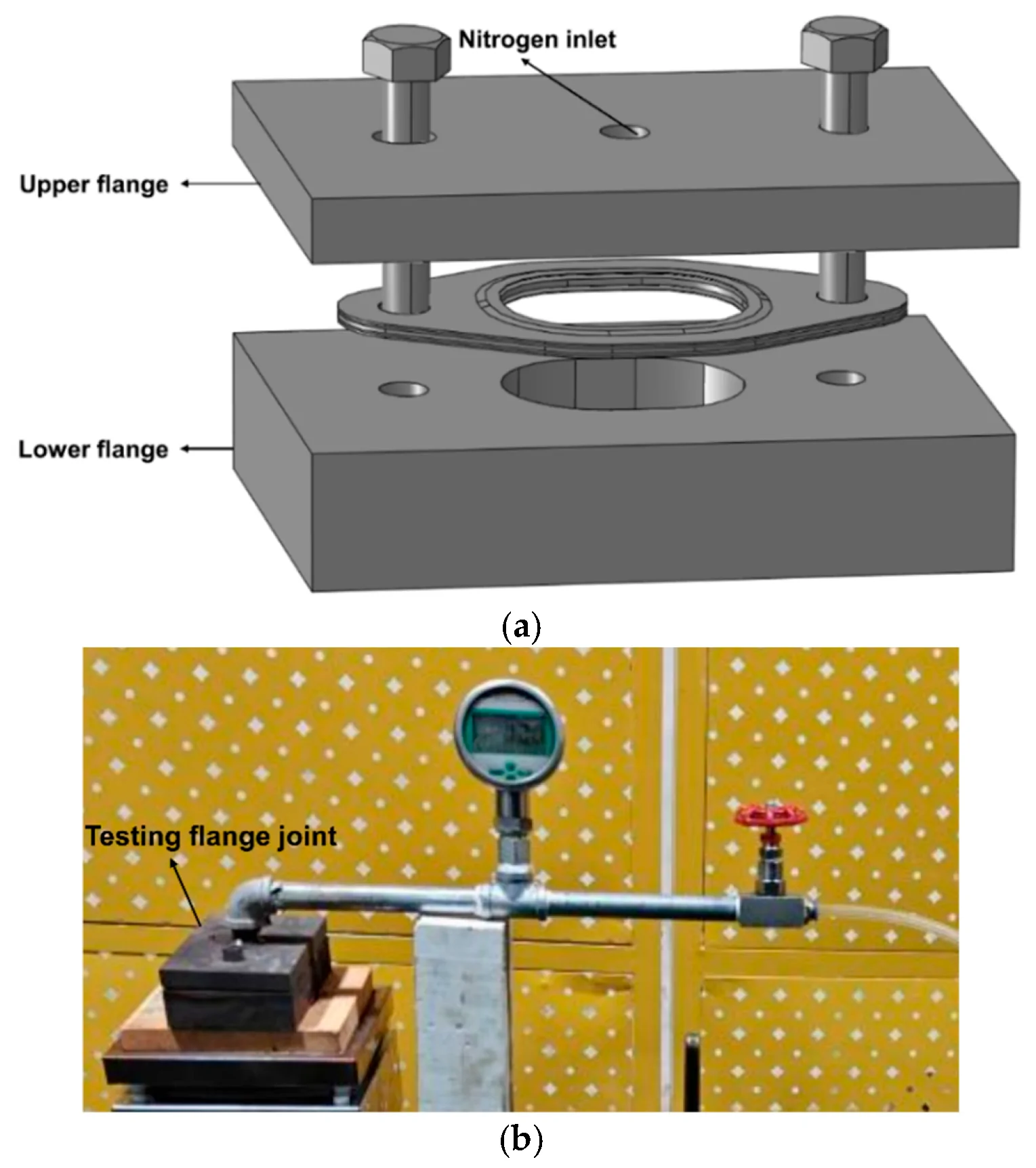
Experimental setup for sealing test: (**a**) testing flange joint and (**b**) testing setup.

In this study, adopting the same thicknesses, the same contact surface roughness, the same materials of the flanges and the same gasket in the front-end flange joint of the studied exhaust manifold, a testing flange joint was designed and fabricated, as shown in Figure 8a, to perform the sealing test. The details of the testing flanges are given in Figure S3 in the Supplementary Information. The testing setup is illustrated in Figure 8b.

### 4.2. Sealing Test of the Flange Joint

The test steps are as follows:

(1) Fix the testing flange joint, and connect the gas supply device and pressure gauge at the inlet of the upper flange.
(2) Pre-tighten the bolts using a torque wrench with a small torque to make initial contact between the sealing surfaces first, and then gradually increase the torque in 2–3 steps until the required preload is reached.
(3) Use a high-temperature furnace to heat the flange joint, gradually heat it to the required temperature with a temperature change rate below 5 °C/min, and use insulation cotton to insulate it.
(4) Open the valve of the gas supply device and adjust the pressure of the dry nitrogen gas to the gas pressure in the exhaust pipe, 0.5 MPa.
(5) Close the gas supply valve and maintain the pressure for 10 min. If the pressure drop meets the nitrogen quality leakage rate requirement of 0.77 mg·m^−1^·s^−1^, the sealing performance of the testing flange joint under the current bolt preload is qualified. Otherwise, increase the bolt preload and go back to step (2) to retest until the pressure drop meets the quality leakage rate requirement, which can be defined as follows:

(3) $$L_{m}=\rho \frac{T_{\mathrm{st}}}{P_{\mathrm{st}}}\frac{V_{C}\cdot P_{\delta }}{T\cdot L\cdot t}$$

where *ρ* is the density of the nitrogen gas, *T*_st_ is the absolute temperature of gas under standard conditions, *P*_st_ is the gas pressure under standard conditions, *V*_C_ is internal volume of the tested system, *P*_δ_ is the pressure drop, *T* is the absolute temperature of the gas, *L* is the length of the sealing ring and *t* is the pressure-holding time.

The critical bolt pre-tightening torques that meet the requirement of the nitrogen quality leakage rate at 500 °C in five repeated tests are shown in Table 3, with an average bolt pre-tightening torque of 51.38 N⋅m and a difference of less than 2% between each test value and the average value.

### 4.3. Sealing Evaluation

**Figure 9:**
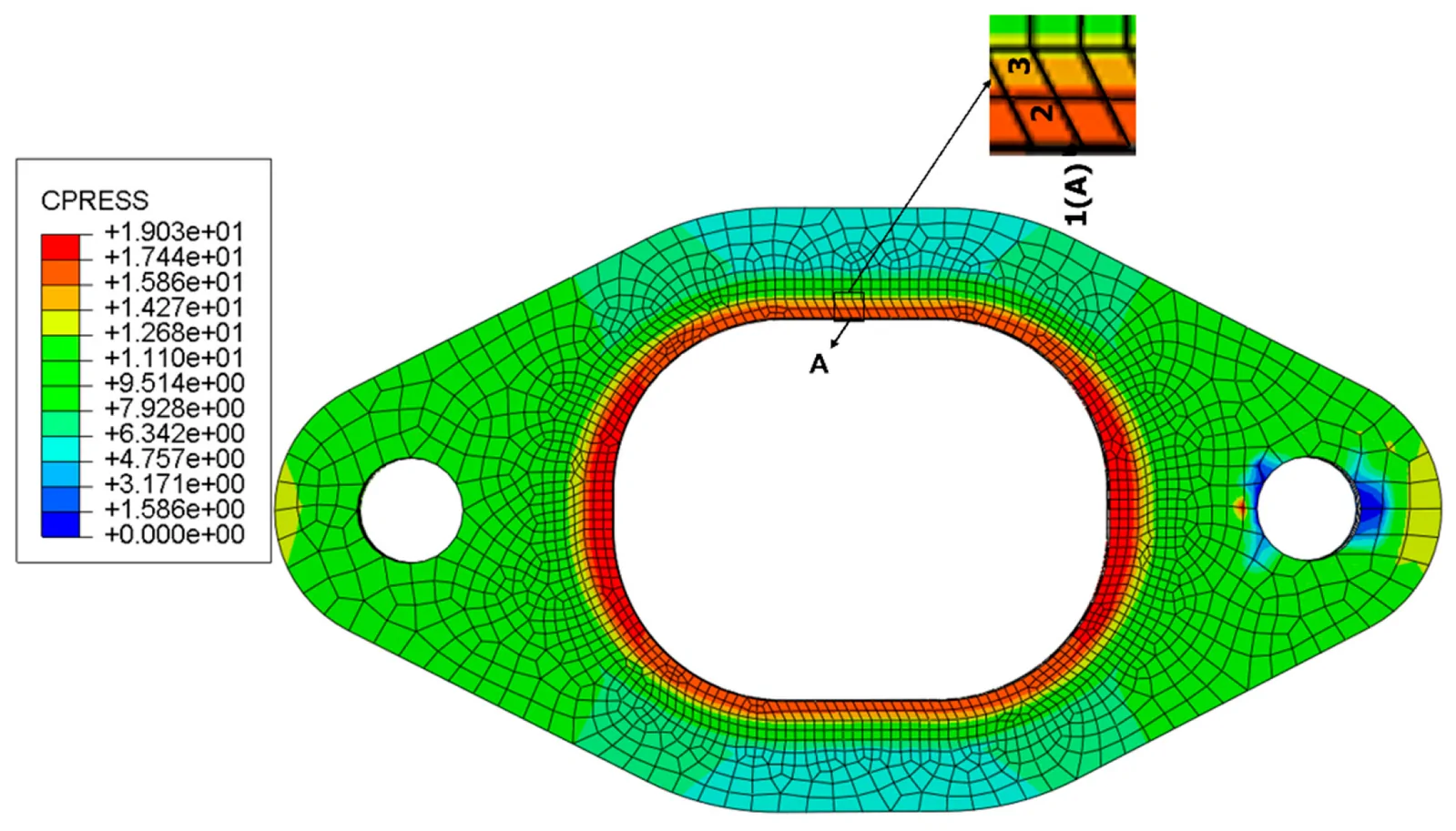
Contact stress distribution on the gasket in the testing joint.

An FE model of the testing joint was developed to calculate the contact stresses at the gasket–flange interface. Figure 9 shows the contact stress distribution of the gasket in the testing flange joint. The contact stress at the minimum contact stress point (point A) on the edge of the sealing ring is 16.2 MPa. The contact stresses of the gasket in the testing flange joint and those in the actual flange joint obtained in Section 3.4 are compared to evaluate the sealing performance of the actual joint, focusing on the contact stresses in the regions near the minimum contact stress point at the sealing ring edge.

**Figure 10:**
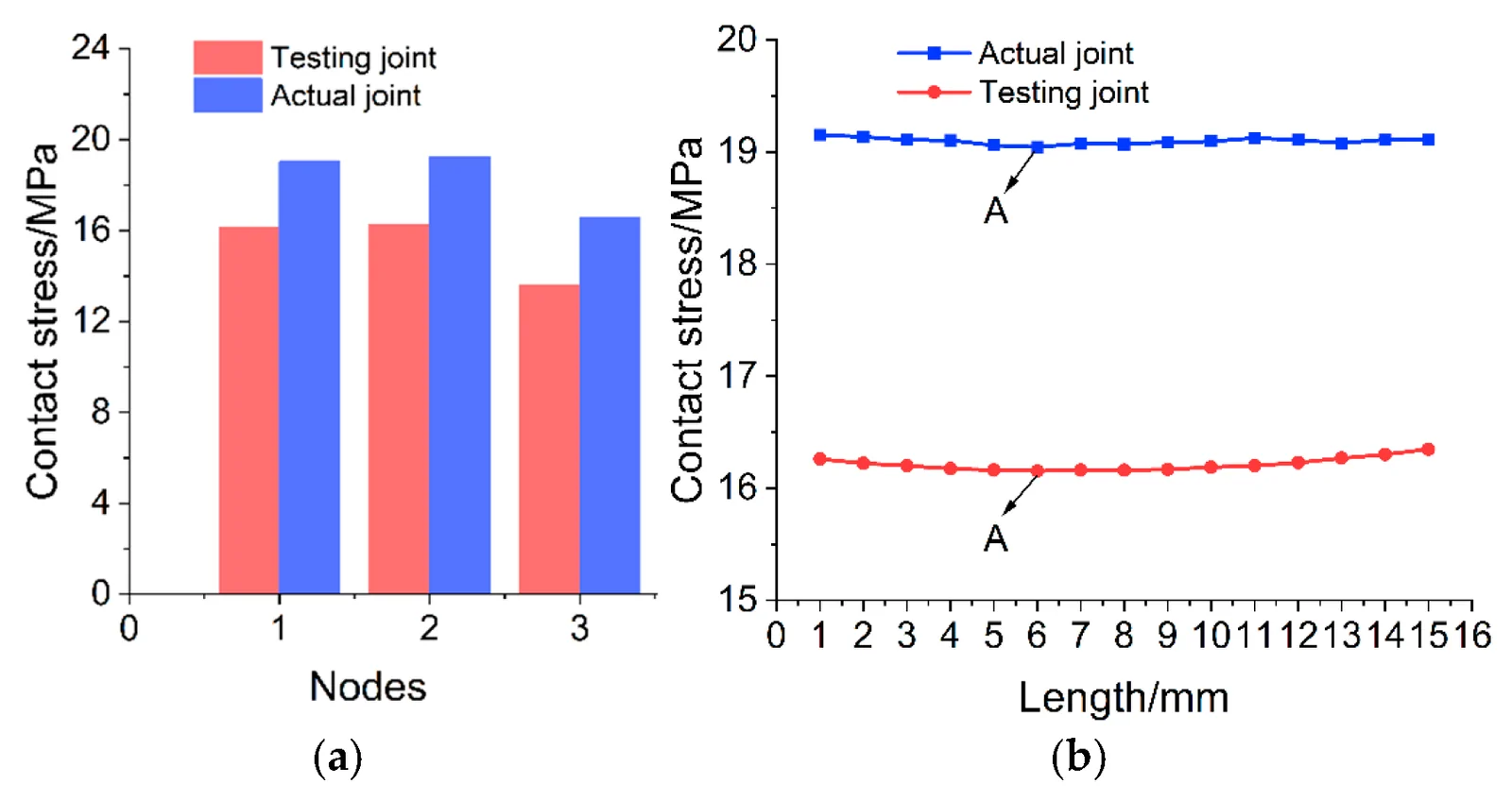
Comparison of contact stresses on the sealing ring of the gasket in the testing joint and the actual joint: (**a**) at the three nodes outward from Point A (Node 1) in the width direction of the sealing ring and (**b**) along the horizontal edge of the sealing ring where Point A is located.

The contact stresses at the three nodes outward from Point A in the width direction of the sealing ring of the gasket in the testing joint and the actual joint are given in Figure 10a in which Point A is located at Node 1 (as shown in Figure 9), and the contact stresses along the horizontal edge of the sealing ring where Point A is located are given in Figure 10b. As shown in Figure 10a, the contact stresses of the gasket in the actual joint over the entire sealing ring width at the minimum contact stress point are greater than those in the testing flange joint and also greater than 16.2 MPa. Figure 10b further confirms that the contact stresses along the horizontal edge of the sealing ring of the gasket in the actual joint are greater than those in the testing flange joint. These results demonstrate that the contact stresses in the key regions in the front-end flange joint of the diesel engine exhaust manifold are higher than the critical contact stress required to meet the leakage rate requirement, thereby ensuring sealing.

## 5. Effect of Material Creep on the Sealing Performance of the Flange Joint

Under long-term high-temperature loading conditions, the creep deformation of materials cannot be ignored because the elastic strain in the contact area will gradually transform into irreversible creep plastic strain and result in a continuous decay of contact stress over time. In this study, the creep curves were obtained through high-temperature creep tests of materials, and the corresponding creep constitutive models were established. The creep test results of the two materials of the gasket (high-nickel alloy and 06Cr19Ni10) showed that their creep strains at 500 °C were very small (see Figure S4), which were ignored here. The FE method was used to analyze the changes of contact stress at the gasket–flange interface of the flange joint after long-term high-temperature service, and the influence of material creep on the sealing performance of the flange joint was evaluated.

A typical metal creep curve can be divided into three stages: unstable creep stage, steady-state creep stage, and rupture stage. The steady-state creep stage lasts for a long time, during which the creep rate is the smallest and remains basically constant, which is the focus of this study. The creep model in the steady-state creep stage can be expressed by the Norton Bailey model [35,36], where its classical power-law form is as follows:

(4) $${\dot{\varepsilon }}^{cr}=A\cdot \sigma^{n}\cdot t^{m}$$

where *A* is the material parameter, *n* is the stress index, and *m* is the time index.

### 5.1. Creep Constitutive Models of the Materials

#### 5.1.1. Creep Constitutive Model of the Cylinder Head Material

**Figure 11:**
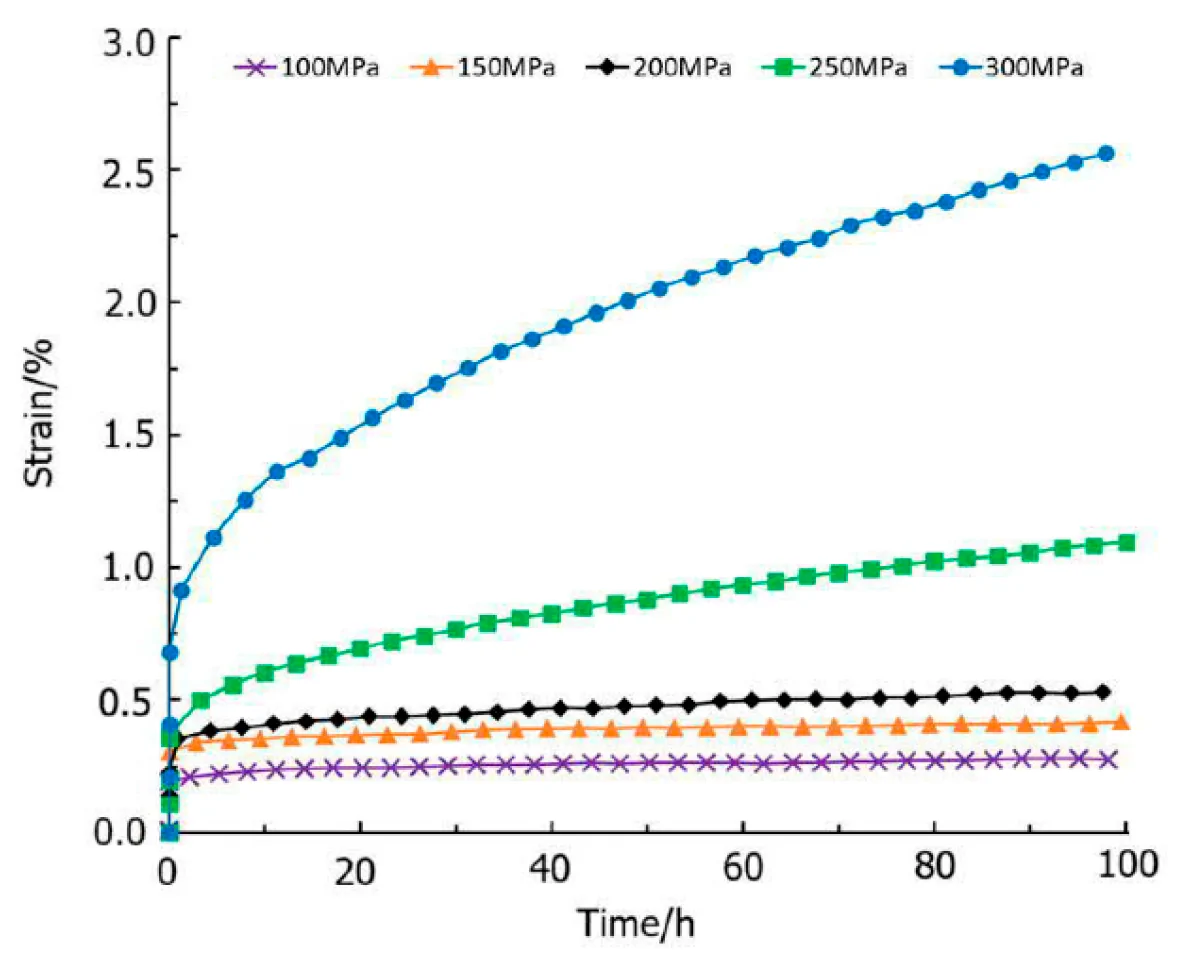
Creep curves of the cylinder head material.

The average temperature of the cylinder head in the exhaust passage area is approximately 400 °C. Therefore, creep tests were conducted on the cylinder head material (CGI) samples at this temperature, with loads of 100 MPa, 150 MPa, 200 MPa, 250 MPa, and 300 MPa, to obtain creep curves (see Figure 11). Linear regression was performed on its steady-state creep rates, and the creep constitutive model of the material was fitted as shown in Equation (3).

(5) $${\dot{\varepsilon }}^{cr}=9.13\times 10^{-13}\sigma^{3.206}$$

#### 5.1.2. Creep Constitutive Model of the Exhaust Manifold Material

**Figure 12:**
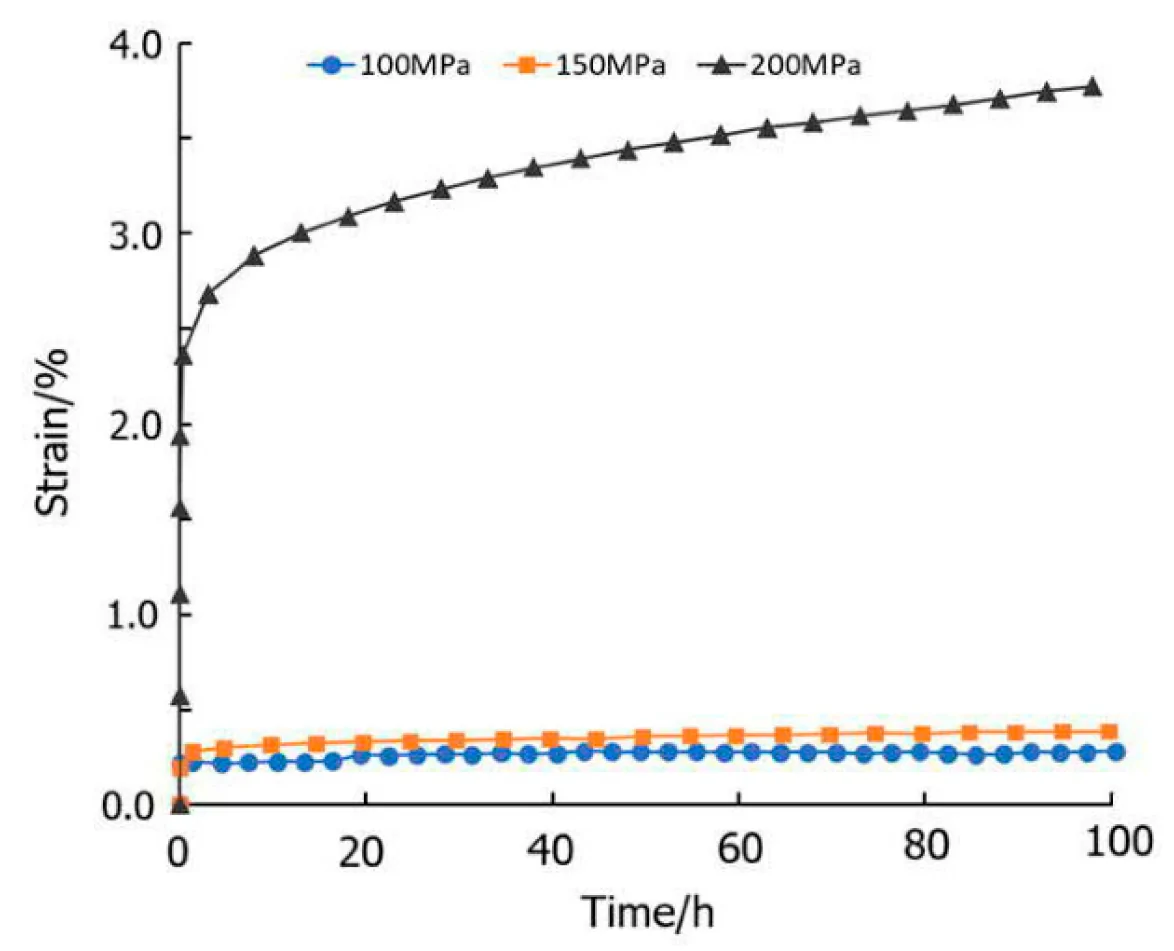
Creep curves of the exhaust manifold material.

The temperature of the exhaust manifold is relatively high, with an average temperature of around 600 °C. Creep tests were conducted on the exhaust manifold material (ZG35Cr25Ni20Si2) sample at this temperature, with loads of 100 MPa, 150 MPa, and 200 MPa, respectively, to obtain creep curves (see Figure 12). The creep constitutive model of the material obtained by fitting is shown below:

(6) $${\dot{\varepsilon }}^{cr}=6.8\times 10^{-18}\sigma^{5.612}$$

#### 5.1.3. Creep Constitutive Model of the Bolt Material

The material of the bolts for the front flange of the exhaust manifold in this study is 42CrMo, and the creep model adopted is shown below [19]:

(7) $${\dot{\varepsilon }}^{cr}=1.64\times 10^{-23}\sigma^{6.9}$$

### 5.2. Effect of Material Creep on Contact Stress at the Interface of the Flange Joint

**Figure 13:**
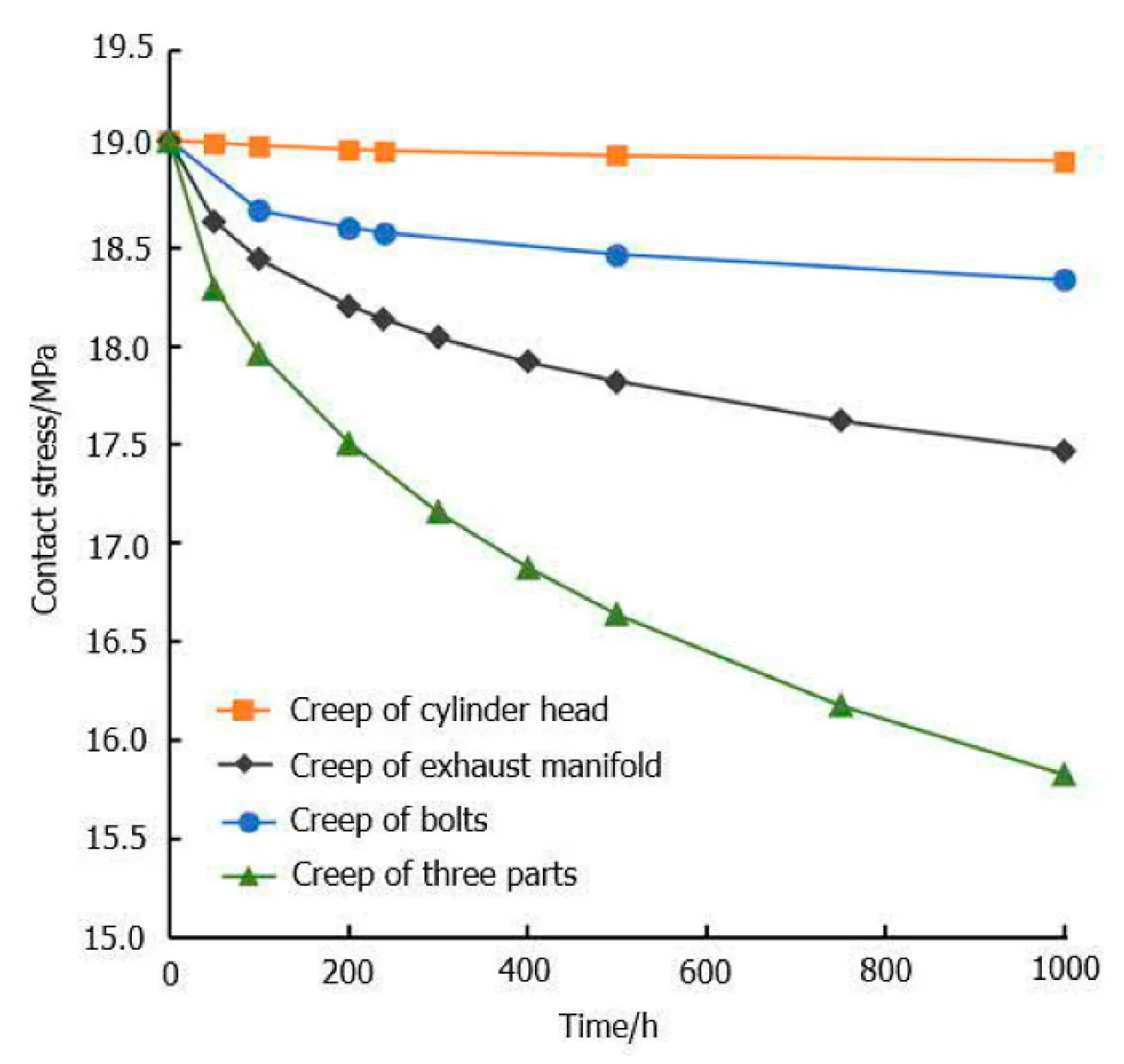
Variation in minimum contact stress on the gasket sealing ring with service time considering the creep of part materials.

The creep constitutive models of each material obtained in Section 5.1 were input into the material properties in the FE model established in Section 3. A creep analysis step was conducted to calculate the variation in the minimum contact stress on the gasket sealing ring with time for the cylinder head, the exhaust manifold, and the bolts, as well as for all three parts experiencing creep. The results are shown in Figure 13. It can be found that the effect of creep of the exhaust manifold on the minimum contact stress is the greatest, followed by the effect of creep of the bolts, and the effect of creep of the cylinder head is the smallest. The reason for this phenomenon is related to the matching of the force and the deformation at the flange joint between the cylinder head and exhaust manifold. The stiffness of the exhaust manifold is relatively low, with the displacement constraints only at the flange joint. Continuous creep elongation/bending deformation occurs at high temperatures, which directly changes the contact stress distributions of the joint surfaces. On the other hand, the overall stiffness of the cylinder head is relatively high, and its contact area with the cylinder block is constrained, as well as the contact constraint with the exhaust manifold flange. Therefore, the deformation caused by the creep of the cylinder head is limited, and the disturbance to the contact stresses of the joint surfaces will be smaller than that of the exhaust pipe. It is estimated that after approximately 750 h of service for all three parts experiencing creep, the minimum contact stress on the gasket sealing ring will decrease to the critical minimum contact stress of 16.2 MPa, and gas leakage may occur at the front flange joint of the exhaust manifold.

## 6. Conclusions

Aimed at the high-temperature sealing design of the front-end flange joint of a high-power diesel engine exhaust manifold, the FE analysis was utilized to determine the thermal contact stress distributions on the joint surfaces in the assembly. An experiment was designed to determine the critical bolt preload required to meet the leakage rate requirement at 500 °C and then obtain the critical minimal contact stresses on the gasket ring through the FE analysis, which is 16.2 MPa. Under the high-temperature working condition, the contact stresses on the gasket of the actual flange joint in the exhaust manifold front end are greater than the contact stresses in the testing flange joint under the critical bolt force for sealing. It can be concluded that the sealing performance of the exhaust manifold front-end flange joint is qualified under this condition.

Considering the effect of material creep on the sealing performance of the flange joint, the creep curves were obtained through the creep tests of the part materials, and the corresponding creep constitutive models were established which were input into the material properties in the FE model. The FE creep analysis results show that the effect of creep of the exhaust manifold on the minimum contact stress is the greatest and the continuous sealing time of the flange joint at the high working temperature was predicted to be about 750 h. These results provide a predictive foundation for the sealing design of the front flange joint of the exhaust manifold.

This study mainly involves predicting and analyzing the sealing performance of the designed front flange joint of the exhaust manifold under the stable high-temperature working condition. The method proposed in this study is also applicable to the sealing performance analysis of other bolted flange joints working at high temperatures. In the future, we will investigate the sealing performance of the flange joint under engine vibration and transient operating conditions, as well as the influence of manufacturing tolerances.

## Figures and Tables

**Table 1:** Temperature values and heat transfer coefficients of different regions.

Region	Combustion Face	Coolant Gallery	Exhaust Port	Inlet	Outer Surface
Temperature [°C]	731	85	650	68	30
Heat transfer coefficient [W·m^−2^·K^−1^]	1500	5700	1250	150	20

**Table 2:** Variation in minimal contact stress on the gasket sealing ring with the element size.

E lement size [mm]	3	2	1.5	1	0.5
Minimal contact stress [MPa]	14.8	18.2	18.6	19.0	19.7

**Table 3:** Critical bolt pre-tightening torques to meet the quality leakage rate requirement.

Test order	1	2	3	4	5	Average
Torque [N⋅ m]	51.18	51.82	52.21	51.37	50.32	51.38

## Data Availability

The original contributions presented in this study are included in the article/Supplementary Material. Further inquiries can be directed to the corresponding author.

## Supplementary Materials

The following supporting information can be downloaded at https://www.mdpi.com/article/10.3390/ma19183830/s1: Figure S1: Comparison of experimental and FE calculated compression–springback curves for the multilayer metallic gasket: (a) at 20 °C and (b) at 200 °C; Figure S2: Influences of sealing ring dimensions on minimum contact stress: (a) height and (b) width; Figure S3: Drawing of the testing flange specimens: (a) the upper flange and (b) the lower flange; Figure S4: Creep curves of the gasket materials: (a) high-nickel alloy and (b) 06Cr19Ni10.
