# Supplementary material for: Sealing Performance Analysis for the Flange Joint at the Front End of a High-Power Diesel Engine Exhaust Manifold
Source: Materials (Basel). 2026 Sep 9;19(18):3830. doi: 10.3390/ma19183830 (PMC13608882; doi:10.3390/ma19183830)
Supplement: Supplementary file 1 [file materials-19-03830-s001.zip › materials-4519322-supplementary.pdf]

Supplementary Information for

Sealing Performance Analysis for the Flange Joint at the Front End of a High-Power Diesel Engine Exhaust Manifold

Qinggele Hua, Jing Ding and Wenjie Qin *

School of Mechanical Engineering, Beijing Institute of Technology, Beijing 100081, China
* Correspondence: qinwj@bit.edu.cn

1. Experimental testing and finite element simulation of compression-springback characteristics of the gasket under lower temperatures

At a joint of bolt flange, a gasket is usually fitted between the two flange surfaces and its deformation in the thickness direction which is often represented by a compression-springback curve which has a significant impact on the contact stresses on flange joint surfaces. In this study, the numerical simulation with experimental verification was used to study the compression-springback characteristics of the multilayer metallic gasket in the front end flange joint of the diesel engine exhaust manifold. Firstly, compression-springback curves at room temperature (20 °C) and 200 °C were experimentally measured by the compression test using a universal testing machine. Subsequently, an FE model simulating the compression test setup was developed and the FE dynamics analysis was implemented to simulate the compression-springback processes. The simulated compression-springback curves at room temperature and 200 °C were compared with the corresponding experimental curves as shown in Figure S1 (a) and Figure S1 (b).

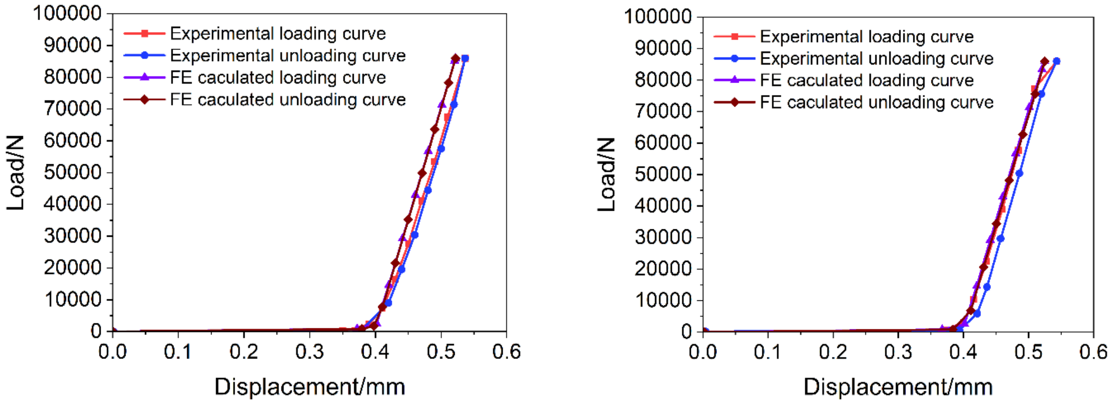

(**a**) (**b**)

Figure S1. Comparison of experimental and FE calculated compression-springback curves for the multilayer metallic gasket: (a) at 20 °C, (b) at 200 °C.

It can be seen that the calculated results are very close to the experimental results. Due to the partial deformation of the testing machine being superimposed on the deformation of the gasket, the measured deformation is slightly greater than the simulation results. Under the room temperature condition, both the loading curve and the unloading curve are basically linear and overlapping, indicating that the gasket deformation is mainly elastic. When the temperature increases to 200 °C, the yield strength of the gasket material decreases, causing the gasket layers to undergo plastic deformation under lower compressive load, and resulting in a slight reduction in the slope of the curves and a

separation between the loading curve and the unloading curve. Comparison with the curve at 500 °C in the main manuscript, it can be found that the slope of the curve further decrease and the separation between the loading curve and the unloading curve significantly increases at 500 °C because of further reduction of the yield strengths of the gasket materials.

2. Effects of height and width of the sealing ring on contact stresses

Parametric finite element analyses were conducted with varying the height and width of the sealing ring in the gasket to investigate their effects on the minimum contact stress. As shown in Figure S2 (a), the minimum contact stress increases with the increasing of the sealing ring height, which is beneficial for sealing. However, if the height is too high, the stress concentration at the transition between the sealing ring and the gasket plane will be more severe, thereby reducing the service life of the gasket. In addition, the increase in minimum contact stress is not very significant when the height is greater than 0.2 mm, so the design sealing ring height of 0.2 mm for the gasket in this study is an optimal compromise for the aforementioned trade-off. As shown in Figure S2 (b), the minimum contact stress on the gasket decreases with the increasing of the sealing ring width, with a particularly sharp decline when the width exceeds 2.5 mm. Although wider sealing rings generally can enhance sealing performance, the optimal width range should be between 2 mm and 2.5 mm to balance the minimum contact stress requirement and the sealing effectiveness.

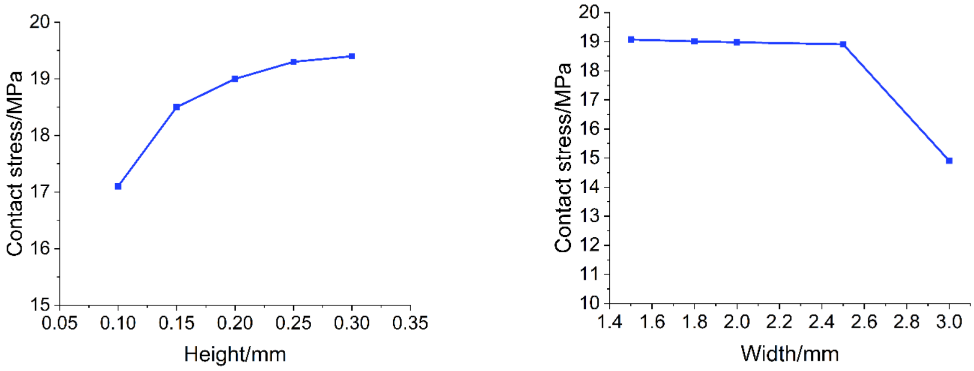

(**a**) (**b**)

Figure S2. Influences of sealing ring dimensions on minimum contact stress: (a) height, (b) width.

3. Specimen dimensions

The dimensions and roughness values of the test flanges are shown in Figure S3.

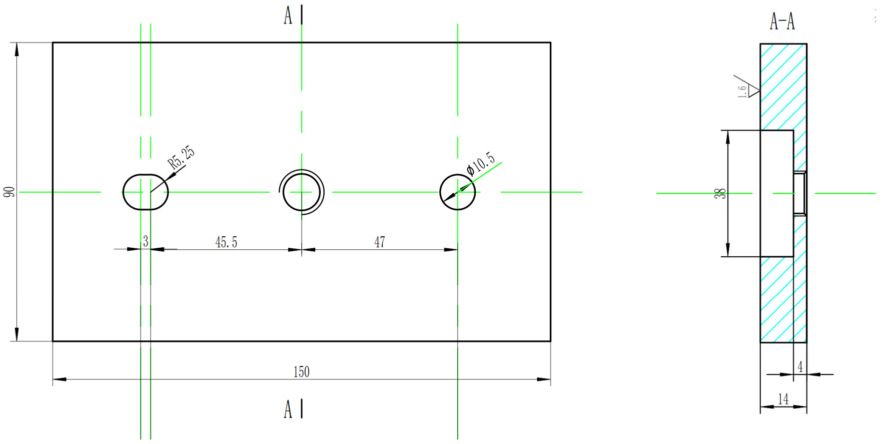

(**a**)

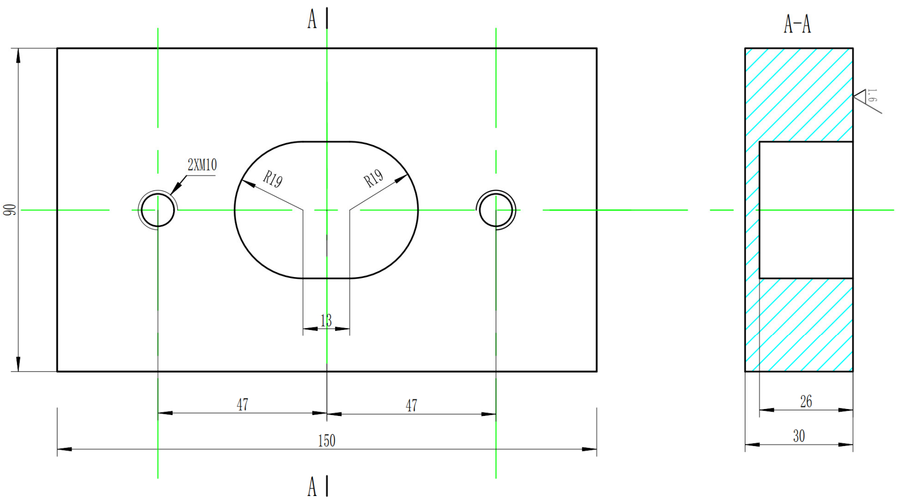

(**b**)

Figure S3. Drawing of the testing flange specimens: (a) the upper flange, (b) the lower flange.

4. Creep curves of the gasket materials

The temperature of the gasket is around 500 ℃. Creep tests were conducted on the two gasket materials (high-nickel alloy and 06Cr19Ni10) at this temperature to obtain the creep curves (see Figure S4). The results show that the cumulative creep strains of both materials during 100 hours are less than 0.1%, so the effect of material creep deformation on the flange sealing performance can be ignored.

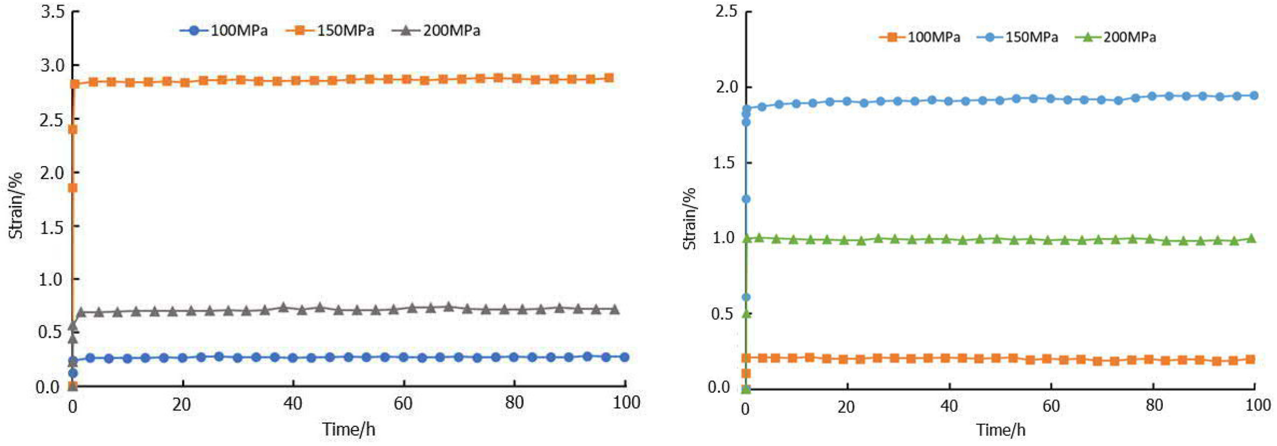

(**a**) (**b**)

Figure S4. Creep curves of the gasket materials: (a) high-nickel alloy, (b) 06Cr19Ni10.
